# A 20-year historical review of West Nile virus since its initial emergence in North America: Has West Nile virus become a neglected tropical disease?

**DOI:** 10.1371/journal.pntd.0009190

**Published:** 2021-05-06

**Authors:** Shannon E. Ronca, Jeanne C. Ruff, Kristy O. Murray

**Affiliations:** 1 Department of Pediatrics, Section of Pediatric Tropical Medicine, Baylor College of Medicine and Texas Children’s Hospital, Houston, Texas, United States of America; 2 William T. Shearer Center for Human Immunobiology, Texas Children’s Hospital, Houston, Texas, United States of America; 3 National School of Tropical Medicine, Baylor College of Medicine, Houston, Texas, United States of America; Instituto Evandro Chagas, BRAZIL

## Abstract

After the unexpected arrival of West Nile virus (WNV) in the United States in 1999, the mosquito-borne virus quickly spread throughout North America. Over the past 20 years, WNV has become endemic, with sporadic epizootics. Concerns about the economic impact of infection in horses lead to the licensure of an equine vaccine as early as 2005, but few advances regarding human vaccines or treatments have since been made. There is a high level of virus transmission in hot/humid, subtropical climates, and high morbidity that may disproportionately affect vulnerable populations including the homeless, elderly, and those with underlying health conditions. Although WNV continues to cause significant morbidity and mortality at great cost, funding and research have declined in recent years. These factors, combined with neglect by policy makers and amenability of control measures, indicate that WNV has become a neglected tropical disease.

## Introduction and history of West Nile virus in North America

West Nile virus (WNV) was first discovered in Uganda in 1937 [1], and for more than 60 years, circulated in an enzootic mosquito-borne transmission cycle throughout Africa, the Middle East, Russia, and Europe, with the predominant strain being lineage 2 [2]. Infections were typically characterized as subclinical or causing mild febrile illness [3]. In the mid-1990s, a new strain of WNV (lineage 1) emerged that resulted in a high proportion of neurological infections, with epizootics occurring in Romania, other parts of Europe, Russia, and Israel [2,4].

In late August of 1999, 2 cases of encephalitis were reported to the New York City Department of Health and Mental Hygiene (NYCDOH) by an infectious disease physician (Dr. Deborah Asnis) in the borough of Queens, prompting an investigation [5]. Similar cases were also quickly identified at neighboring hospitals, and the NYCDOH requested assistance from the Centers for Disease Control and Prevention (CDC) to help identify the cause. Based on patient and family interviews and environmental inspections, it became apparent that the most likely pathogen was mosquito borne. Initial testing of sera and cerebrospinal fluid (CSF) from a subset of suspect patients were found to be positive for IgM antibodies against St. Louis encephalitis virus (SLEV) by monoclonal antibody capture-enzyme linked immunosorbent assay (MAC-ELISA) at CDC, leading to swift implementation of mosquito control measures [6].

While massive die-offs of American crows (family Corvidae) were initially thought to be linked to the human outbreak, the New York state pathobiologist reported that the deaths were related to mass poisonings. Approximately 2 weeks after the launch of the investigation by NYCDOH, captive exotic birds at the Bronx Zoo began to die with encephalitis determined to be the cause of death as diagnosed by the zoo veterinary pathologist, Dr. Tracey McNamara [6]. Brain tissues from these birds were sent to the United States Department of Agriculture National Veterinary Services Laboratories (NVSL), with viral isolates then sent on to CDC for sequencing [7]. Sequencing initially revealed a strain most likely related to WNV, initially referred to as WNV-like then later confirmed to be WNV lineage 1, most closely related to the 1998 Israel strain [7]. Simultaneously, researchers isolated virus from brains of patients who died from encephalitis during the outbreak and identified a Kunjin/West Nile-like flavivirus based on sequencing, further confirming the etiology [8]. Crow deaths were then confirmed to be caused by WNV [6]. By October, 15 horses had developed encephalitis on Long Island in New York and were found to be positive for WNV. Finally, virus was isolated from *Culex pipiens* mosquitoes, providing evidence for the presumed vector for virus transmission [9]. Before 1999, WNV had never been detected in the Western Hemisphere.

Retrospective IgM ELISA testing of sera and CSF from patients with encephalitis or meningitis who were previously tested for SLEV were confirmed positive for WNV infection, even in those who previously had negative or equivocal SLEV results [6,10]. Ultimately, the New York City (NYC) outbreak in 1999 led to 62 confirmed cases, including 7 deaths [5]. Toward the end of the outbreak, a large household-based cluster serosurvey was conducted in the area of Queens that was most affected [11]. Investigators identified a weighted (adjusting for clusters) seroprevalence of 2.6% for WNV infection. Based on reported signs and symptoms of study participants, researchers extrapolated that approximately 80% of those infected were asymptomatic, approximately 20% developed uncomplicated febrile illness, and only 1 out of 140 developed the more severe disease process of encephalitis or meningitis, later coined “West Nile neuroinvasive disease” (WNND). Based on this study’s estimates, approximately 8,200 were infected with WNV in NYC during the initial outbreak [11].

It is still unknown as to how the virus was introduced into NYC. Plausible theories include introduction of infected mosquitoes through shipping or airline cargo, infected migratory birds from Europe, infected imported birds or domestic animals, intentional introduction (bioterrorism), or a viremic person. While humans are considered dead-end hosts, the latter theory could be possible if the infected person was severely immunocompromised.

## Geographic emergence

Following the outbreak in 1999, there was uncertainty about the virus’s ability to overwinter and then disperse to new geographical areas. Considering the high percentage of deaths in corvids, avian mortality surveillance was established at CDC as a means of early detection of virus activity and as a monitoring tool for geographic spread [12–14]. Ultimately, avian mortality surveillance was merged with human, horse, and vector surveillance into a new surveillance tool called ArboNET, with real-time mapping links provided by the US Geological Survey [15]. As predicted, avian mortality surveillance became an effective tool in early detection as it moved into new geographic areas, with detection of bird deaths often preceding the detection of positive mosquitoes and human cases [12,16–18].

In the first year after the initial outbreak in NYC, WNV remained isolated to the northeast, with 21 human cases of WNV infection identified in New York, New Jersey, and Connecticut [19]. In 2001, WNV spread geographically along the eastern coast, with 66 cases identified in 10 states between Massachusetts and Florida. With the southern spread, the virus was detected in *Culex quinquefasciatus* populations, creating concern for further epidemic potential as seen with SLEV, a related flavivirus [20–22]. Still, the spread and degree of epidemic transmission in 2002 was unprecedented, with 4,156 human cases of WNV infection in the US, including 284 deaths [19], reported as far west as Texas and Montana and as far north as the provinces of Quebec and Ontario, with 414 Canadian cases reported [23]. Epizootic transmission was also high, with more than 15,000 equine cases reported [24]. During this epidemic, it became quickly evident that WNV could be transmitted person to person through viremic blood donations, organ transplant, transplacental movement, and breast milk [25]. This prompted an emergency response to develop blood donor screening to prevent contaminated blood supply, which was implemented in 2003 [26,27]. By 2003, the virus was found in *Culex tarsalis*, and the unprecedented spread continued, affecting 45 US states, with 9,862 cases and 264 deaths reported [19]. It is important to note that the total number of cases in the US in 2003 was somewhat inflated compared to prior years since CDC requested non-neuroinvasive disease cases (i.e., West Nile fever [WNF]) to also be reported to public health [13]. Similar to the US, WNV had continued its spread in Canada, with their health website reporting 5 provinces with confirmed autochthonous infections and 8 provinces/territories reporting a total of 1,481 human cases. Serologic evidence of the virus had been reported in horses and avian hosts in some countries in Latin America and the Caribbean; however, human cases of infection were unexplainably rare [28].

**Fig 1:**
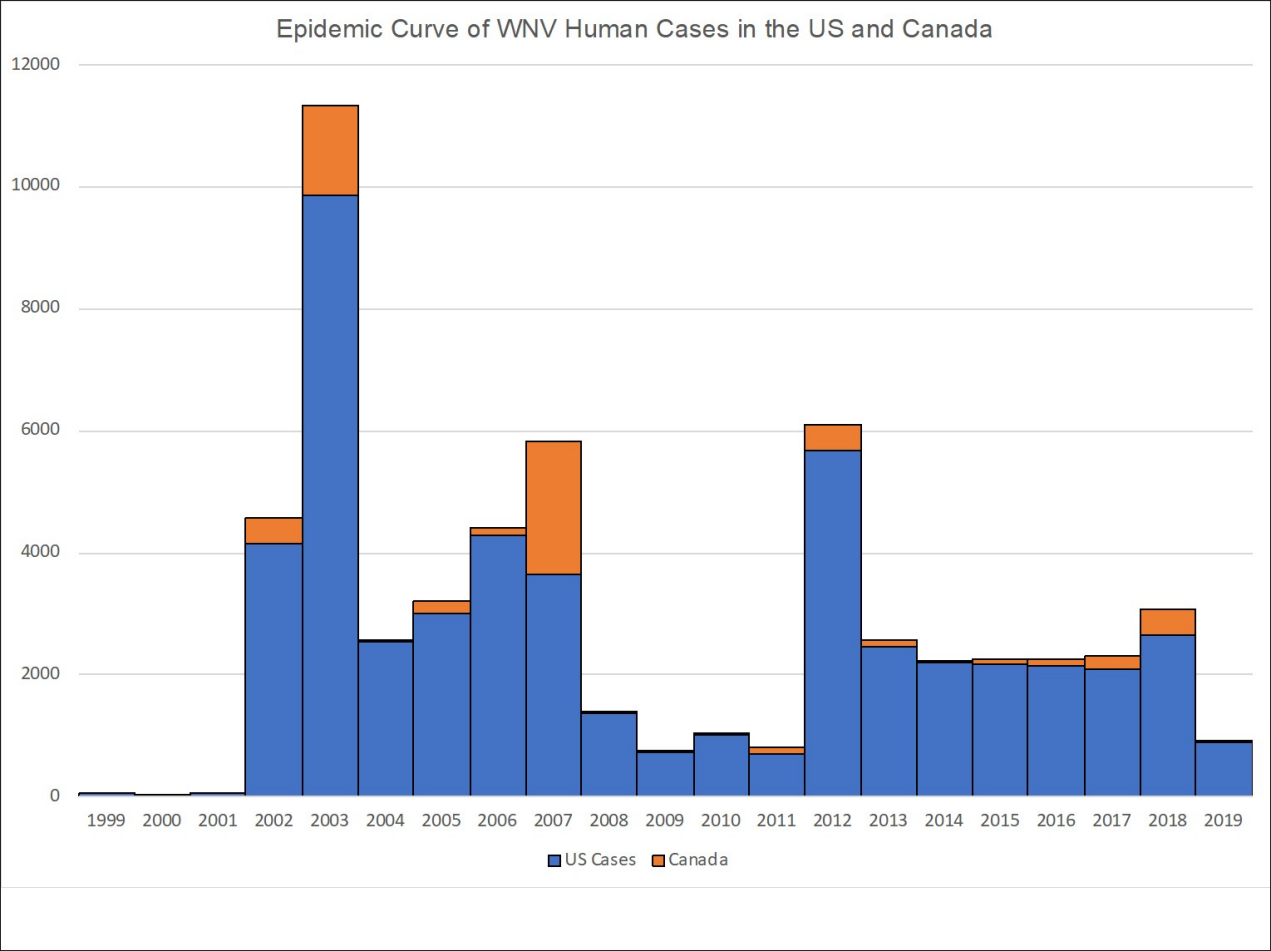
Epidemic curve of WNV human cases in the US and Canada, 1999–2019. WNV, West Nile virus.

By 2004, the WNV epizootic had spread along the West Coast of the US, and by 2012, all 48 continental states and the District of Columbia had reported a locally acquired human case [29]. Between 2002 and 2007, WNV was at peak epidemic levels in both the US and Canada (Fig 1), then declined dramatically between 2008 and 2011. In 2012, an unexpected epizootic occurred with record numbers of cases in Texas [30], Louisiana, Oklahoma, Arkansas, Mississippi, and Alabama [19]. High numbers of cases (>2,000 annually) continued to be reported between 2013 and 2018, supporting the high potential for epizootic outbreaks for the foreseeable future. Drivers of epizootic transmission, including mosquito abundance, bird population turnover, and climate conditions and change, need to be further explored in order to predict future outbreaks.

### Phylogenetics

**Fig 2:**
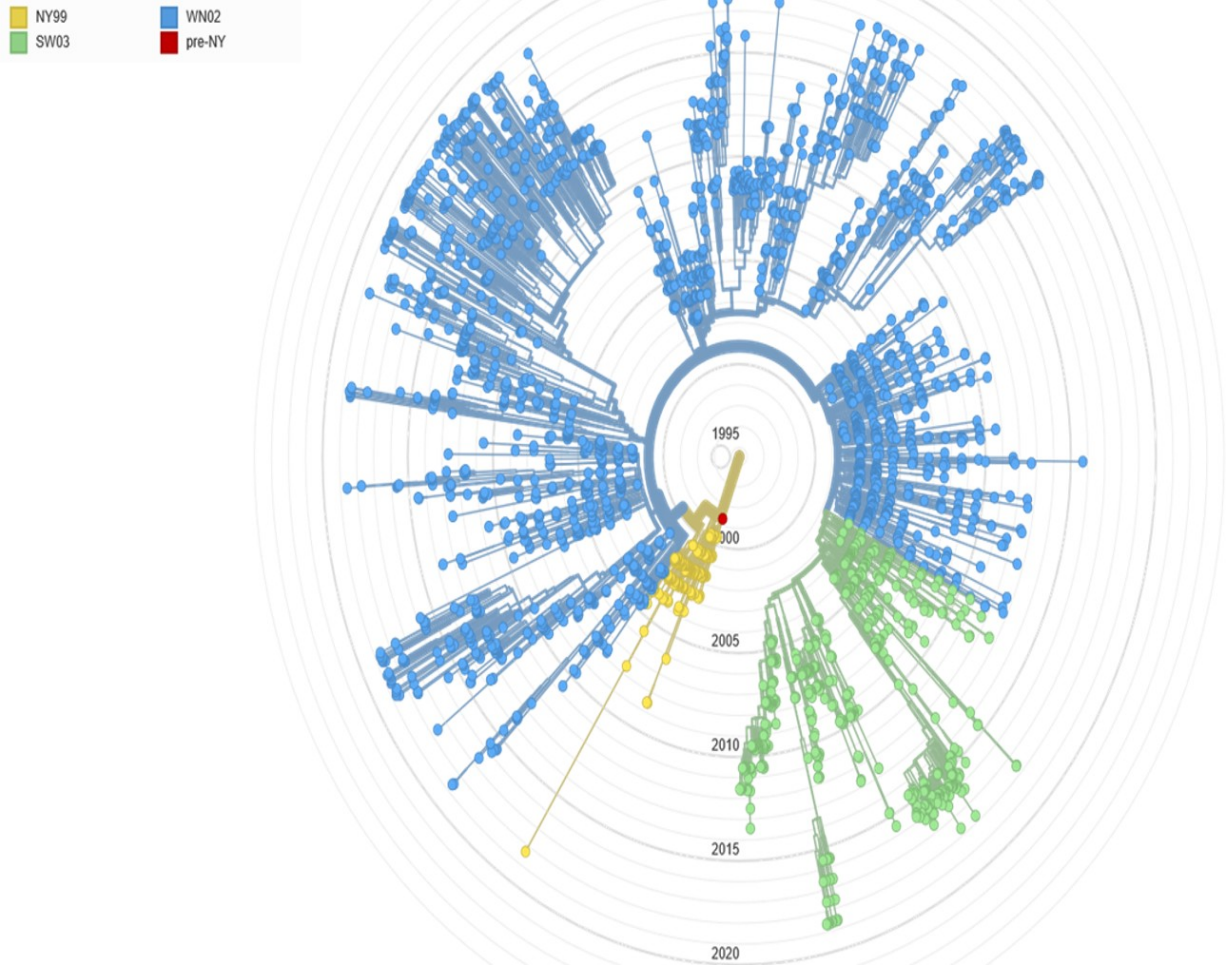
WNV strains over time. *This image was reproduced with permission from N*. *Grubaugh from the data available at* *https*:*//nextstrain*.*org/WNV/NA* [21,45]. WNV, West Nile virus.

A shift in the circulating strain of WNV was observed between the original outbreak in New York in 1999 and subsequent outbreaks since 2002 [2,20,31–34]. The NY99 strain is highly neurovirulent in mice [35]. When passaged in hamsters, this strain also created a chronic kidney infection, which was then replicated in mice using the hamster-passaged strains [36–39]. A different strain, WN2002, with 2 nucleotide differences, was identified during the 2002 outbreaks and remains in circulation today [21,34]. As described in a 20-year analysis of available WNV genotypes by Hadfield and colleagues, WN02, in conjunction with SW03, has displaced the NY99 strain in the US [20,21] (Fig 2). Some studies suggest that species of *Culex* mosquitoes more efficiently transmit WN02 viral strains in comparison to the NY99 strains [40,41], but not all studies agree [42–44]. In mouse models, this WN02 strain has been found to create flaccid paralysis, similar to what is seen with human disease [35]. Continued genotype and phenotype evaluation of strains isolated from endemic regions is necessary to ensure that the strains used in animal models to evaluate vaccines and therapeutics are appropriate.

### Infection burden of WNV in humans, USA

Over the 20-year existence of WNV in the US (between 1999 and 2019), a total of 51,702 cases of WNV have been reported to CDC’s ArboNET, including 25,227 (48.8%) WNND and 2,376 (4.6%) deaths [45]. Based on Mostashari’s original estimate of 1 WNND case for every 140 infections, these 25,227 WNND cases would suggest nearly 3.5 million infections in the US to date. However, additional serological studies indicate that this extrapolation is likely an underestimate [46–48]. A study published in 2013 by Petersen and colleagues evaluated the cumulative incidence of WNV in US adults from 1999 to 2010 by applying Carson and colleagues’ methods [47] and determined that 3 million adults were likely infected with WNV during this time frame [49]. Since approximately 40% of all WNND cases occurred since Petersen’s study and their study did not reflect infections of children, our team updated the estimated number of cases in all age groups from 1999 to 2016 using the methods of Petersen, but including children as estimated by Mandalakas and colleagues [48]. In this study, we estimated nearly 7 million WNV infections in the continental US. This is likely an underestimate since the few studies that evaluate testing frequency have determined that only approximately 40% of cases that meet criteria for WNND are tested upon presentation to a healthcare facility [50,51].

Understanding the true burden of WNV is critical. Certain populations are especially vulnerable to infection, including the homeless and others affected by socioeconomic determinants [52–56], and severe disease, including the elderly and those with underlying medical conditions [5]. Epidemiological studies describe severe and chronic sequelae due to WNV infections, especially in individuals with WNND where up to 40% of patients fail to return to their baseline health status [57]. Understanding the true burden would also provide valuable data for evaluating public health policies for disease control and prevention as well as determining the need for investment in clinical trials for vaccine development and therapeutics.

### The cost of West Nile

With the high disease prevalence and endemicity of WNV in North America, it’s necessary to understand the affiliated burden to the healthcare system and impact on the economy. Zohrabian and colleagues determined the short-term costs of WNV in Louisiana from June 2002 to February 2003 to equal US$10.9 million, with US$4.4 million in medical costs and US$6.5 million in nonmedical costs such as loss of productivity [58]. In a similar study, Barber and colleagues [59] estimated costs related to the 2005 WNV outbreak in Sacramento County, California. This outbreak of 163 people cost about US$2.98 million, including vector control (approximately US$702,000), medical costs for treatment, and productivity loss. They determined only 15 cases of WNND needed to be prevented for vector control measures to be considered cost-effective [59]. A study in Quebec, Canada determined the costs to the region from 2012 to 2013 [60]. In this study, 90 patients were evaluated retrospectively, and median costs were US$21,332 per encephalitis patient, US$8,124 per meningitis patient, and US$192 per WNF patient. Overall, they estimated that approximately US$1.7 million was spent on 124 symptomatic cases in 2012 and US$430,000 on 31 symptomatic cases [60]. Taking it a bit further, Staples and colleagues estimated both the short- and long-term costs related to an outbreak involving 80 cases in Colorado in 2003. It is important to highlight the ranges of costs for these patients, as acute care costs ranged from approximately US$4,000 to US$325,000 for encephalitis cases, approximately US$5,000 to US$283,000 for acute flaccid paralysis (AFP), approximately US$1,000 to US$15,000 for meningitis, and approximately US$500 to US$24,000 for WNF, as this confirms the extreme and unique scenarios that patients can experience. When evaluating long-term costs, the ranges are approximately US$0 to US$24,000 for encephalitis cases (likely due to a higher mortality rate than others), approximately US$600 to US$440,000 for AFP, approximately US$0 to US$261,000 for meningitis, and approximately US$0 to US$41,000 for WNF. These long-term costs include medical appointments and equipment, medications, long-term care, and loss of productivity, but we lack information to tease out how parameters of acute infection, such as length of hospital stay, may affect long-term costs. Using their calculations from these patients, they estimated the total cost of WNV from 1999 to 2012 in the US to equal approximately US$778 million, with a confidence interval ranging from US$673 million to US$1.01 billion [61]. Annually, this equates to US$56 million lost to WNV.

A study of vaccine cost-effectiveness from Zohrabian and colleagues stated that a universal vaccine program in the US is unlikely to result in savings [62]. However, this study occurred early in the introduction of WNV and evaluated case burdens from 1999 to 2004. At that time, approximately 16,000 total cases and 7,000 cases of WNND had occurred. The major outbreak of 2012 was forthcoming, but unanticipated. The authors did discuss that risk for infection, probability of symptomatic illness, and vaccination cost are critical to the evaluation of cost-effectiveness. Since this study was published in 2006, these factors have changed. Additionally, we must consider the needs for vaccines in regions with low population density where mosquito control is less feasible over large land areas.

It is difficult to interpret whether this study directly impacted the future progress of WNV vaccines in development or the investment in commercializing those available. Over 10 years later in 2017, Staples and colleagues [61] evaluated the cost-effectiveness of targeted vaccination and concluded that age-based vaccination may be the most cost-effective method to combat WNV, but we have yet to see a vaccine reach the market to evaluate this to its full potential.

### Vaccine development

The rapid dissemination of WNV throughout North America in the early 2000s and the high cost of morbidity sparked intense interest in the development of a WNV vaccine for both humans and other animals, especially horses. An inactivated whole-virus vaccine was quickly developed and was licensed for veterinary use in 2003 [64]. A variety of equine vaccines were approved in the coming years, including the first DNA vaccine to be licensed by the US Department of Agriculture (USDA) in 2005 [64] (press release: https://www.cdc.gov/media/pressrel/r050718.htm).

A number of human vaccine candidates remain in preclinical stages of development [63]. To date, 9 clinical trials evaluating human WNV vaccine candidates have been registered on ClinicalTrials.gov, with only 6 individual agents investigated (Table 1). Thus far, no human trial has progressed past Phase II.

**Table 1:** Vaccine trials for WNV.

VACCINES								
Title	NCT Number	Dates	Phase	Status	Interventions	Number Enrolled	Sponsor/ Collaborators	Publications
Safety of and Immune Response to a West Nile Virus Vaccine (WN/DEN4-3’delta30) in Healthy Adults	NCT00094718	Posted: 10/22/2004 Completed: 4/2005	1	Completed	WN/DEN4-3’delta30 vs Placebo (vaccine diluent)	56	Johns Hopkins Bloomberg School of Public Health, NIAID*	PMID: 23968769
Safety of and Immune Response to a West Nile Virus Vaccine (WN/DEN4delta30) in Healthy Adults	NCT00537147	Posted: 9/28/2007 Completed: 6/2009	1	Completed	WN/DEN4delta30 vs Placebo (vaccine diluent)	26	Johns Hopkins Bloomberg School of Public Health, NIAID*	
Evaluating the Safety and Immunogenicity of a Live Attenuated West Nile Virus Vaccine for West Nile Encephalitis in Adults 50 to 65 Years of Age	NCT02186626	Posted: 7/10/2014 Completed: 7/2016	1	Completed	WN/DEN4delta30 vs Placebo (vaccine diluent)	28	NIAID*	PMID: 28077583
Vaccine to Prevent West Nile Virus Disease	NCT00106769	Posted: 3/30/2005Completed: 1/15/2008	1	Completed	VRC-WNVDNA017-00-VP	15	National Institutes of Health Clinical Center, NIAID*	PMID: 18190252
Phase I Study of West Nile Virus Vaccine	NCT00300417	Posted: 3/8/2006 Completed: 12/28/2007	1	Completed	VRC-WNVDNA020-00-VP	30	National Institutes of Health Clinical Center, NIAID*	PMID: 21398392
Safety and Immunogenicity of ChimeriVax-WN02 West Nile Vaccine in Healthy Adults	NCT00442169	Posted: 3/1/2007 Completed: 4/2009	2	Completed	ChimeriVax-WN02 vs Placebo (normal saline)	208	Sanofi Pasteur	PMID: 21148499
Safety and Immunogenicity Study of ChimeriVax West Nile Vaccine in Healthy Adults	NCT00746798	Posted: 9/4/2008 Completed: 12/2009	2	Completed	ChimeriVax-WN02 vs Placebo (normal saline)	479	Sanofi Pasteur	PMID: 22959989
Safety Study of HBV-002 West Nile Vaccine in Healthy Adults	NCT00707642	Posted: 7/1/2008 Completed: 6/2009	1	Completed	WN-80E	25	Hawaii Biotech, Inc	Patent listing: http://patft.uspto.gov/netacgi/nph-Parser?Sect1=PTO1&Sect2=HITOFF&d=PALL&p=1&u=%2Fnetahtml%2FPTO%2Fsrchnum.htm&r=1&f=G&l=50&s1=10039820.PN.&OS=PN/10039820&RS=PN/10039820
Phase 1 Trial of Inactivated West Nile Virus Vaccine	NCT02337868	Posted: 1/14/2015 Completed: 12/16/2016	1	Completed	HydroVax-001 vs Placebo	96	NIAID*	PMID: 30661836

*NIAID, National Institute of Allergy and Infectious Diseases.

WNV, West Nile virus.

The first agent to undergo a Phase I clinical trial was a live attenuated West Nile–Dengue chimeric vaccine. Three Phase I studies for this vaccine candidate were registered on ClinicalTrials.govhttp://clinicaltrials.gov/, in 2004, 2007, and 2014. This chimeric vaccine is based on the DENV-4 vaccine candidate rDEN4delta 30, with the prM and E protein genes replaced by those of the WNVNY99 strain [65]. Minimal adverse effects were observed during all 3 Phase I trials. The 2004 and 2007 studies demonstrated seroconversion in approximately 75% of vaccinated participants 18 to 50 years of age after one 10^3^ or 10^4^ plaque-forming unit (PFU) dose. A higher dose of 10^5^ PFU resulted in a lower rate of seroconversion (55%) but was increased to 89% after a 6-month booster [65]. In the 2014 study of adults aged 50 to 65, 19/20 (95%) subjects seroconverted after one 10^4^ PFU dose. A 6-month booster was again administered but did not result in a profound increase in antibody titer and was therefore believed to be unnecessary [66].

In 2005 and 2006, 2 Phase I trials began to investigate the use of a DNA vaccine similar to the one approved for use in animals. Nucleic acid vaccines encode for specific antigenic subunits of the pathogen and recruit the host’s cells to produce these antigens. This is an attractive technology because it can produce long-lasting immunity without introducing any part of the pathogen besides the antigen, thereby ensuring a specific immune response without risk of reactivation of attenuated live virus [67]. Both of these vaccine candidates coded for the same prM and E proteins used in the chimeric candidate described above, although one incorporates a modified version of the gene promotor component [67,68]. Both trials report mild adverse effects related to the vaccines and favorable immune responses. The later version, which incorporated the modified gene promoter, did appear to elicit a stronger cellular and humoral immune response [68].

Additionally, a recombinant subunit vaccine with adjuvant was registered for clinical trial in 2008 and patented by Hawaii Biotech in 2018, although no report of the findings in a human clinical trial have been published. Another, listed in 2015, examined an inactivated whole-virus vaccine, HydroVax-001, which was found to be generally well tolerated at both 1 mcg and 4 mcg concentration in a 2-dose sequence. As expected with an inactivated virus vaccine, no participant was found to be viremic when tested 4 days after each administration, but it failed to produce seroconversion via PRNT_50_ at the lower dose, and only 31% seroconverted after the higher dose. The ELISA-specific response was slightly better, reaching 41% seroconversion after the second 1 mcg dose and 75% after the 4 mcg dose [69].

Only 1 vaccine candidate has entered Phase II clinical trials. This is the ChimeriVax-WN002 agent, another live attenuated chimeric virus. This virus also contains the prM and E protein genes from NY99 but is built on the scaffold of the Yellow fever 17D virus. A favorable safety profile among adults was demonstrated in a Phase I trial [70], and in older adults in 2 Phase II trials [63,71]. Viremia was low and not associated with adverse events among any age group. At least a 4-fold increase in antibody titers was observed in >90% of all treated participants in both Phase II trials [63,71].

These preliminary trials demonstrated that all current vaccine candidates were generally well tolerated and immunogenic to varying degrees, yet progress toward licensure for human use stalled over time. Hurdles to moving these trials forward may include financial concerns and regulatory requirements, which may be difficult to justify due to the large numbers of enrolled volunteers and resources needed when outbreaks of WNV are sporadic.

### Therapeutics

Paralleling the delay in vaccine approval, there are also no specific therapeutics to treat WNV infections. A number of case series and case studies describe the use of interferon, ribavirin, and corticosteroids [72–77], but reports are inconclusive, and no clinical trials have formally evaluated these options. In fact, only 3 agents have been registered for clinical trials in the US. A Phase I/II trial registered in 2003 examined Omr-IgG-am, which is a high anti-WNV titer intravenous immunoglobin (IVIG) compound that is approved for use in Israel. This trial had a low and high dose cohort and was controlled using an active placebo IVIG (Polygam S/D) and a passive placebo of normal saline. No significant difference was noted between the treatment, active placebo, or passive placebo groups regarding adverse effects or 90-day outcome. Unfortunately, this trial experienced multiple challenges enrolling participants and obtaining both the study drug and the active placebo, so it was ended before target enrollment was reached, and the higher dose treatment arm was abandoned [78].

Then in 2004, Sarepta Therapeutics registered a trial to investigate the phosphorodiamidate morpholino oligomer (PMO) agent AVI-4020. PMOs are nucleic acid analogs which, in the context of WNV, can be used to block translation of viral proteins, thereby preventing viral replication [79]. As of this writing, the company has established that AVI-4020 is present in the CSF of healthy volunteers up to 18 hours after a single dose [80], but no reports about the trial examining the use in WNND have been published.

The final agent registered for clinical trial in the US is MGAWN1, a monoclonal antibody. A 2007 to 2009 Phase I trial found that this drug was generally well tolerated by 40 healthy participants after a single IV dose ranging from 0.3 mg/kg to 30 mg/kg. One participant was found to have developed anti-MGAWN1 antibodies 3 months after exposure to the drug. The authors report that this may have affected the efficacy of the drug for this participant, but that since a single dose appears to provide sufficient coverage for the duration of acute illness, this is not thought to pose a large risk for sensitivity reactions [81]. Given the apparent success of the Phase I trial, a Phase II trial investigating the same drug began in 2009. However, like the OMR-IgG-am trial, it was terminated early due to low enrollment. All studies are summarized in Table 2.

**Table 2:** Treatment trials for WNV.

TREATMENT								
Title	NCT Number	Dates	Phase	Status	Interventions	Number Enrolled	Sponsor/ Collaborators	Publications
IVIG—West Nile Encephalitis: Safety and Efficacy	NCT00068055	Posted: 9/8/2003 Completed: 12/2006	1, 2	Completed	Omr-lgG-am vs standard IVIG (Polygam S/D) vs Placebo (normal saline)	62	NIAID*	PMID: 31625835
Omr-IgG-am for Treating Patients With or at High Risk for West Nile Virus Disease	NCT00069316	Posted: 9/23/2003 Completed: 6/27/2007	2	Completed	Omr-lgG-am vs standard IVIG (Polygam S/D) vs Placebo (normal saline)	2	National Institutes of Health Clinical Center	Cited in an observational study of WNV neurologic outcomes (PMID: 24884681), no published reports of use for treatment of WNV identified.
An Exploratory Study of AVI-4020 in Patients With Possible Acute Neuroinvasive West Nile Virus (WNV) Disease	NCT00091845	Posted: 9/24/2004 Completed: 11/2004	1	Terminated	AVI-4020 Injection (multiple doses)	50 (target enrollment, actual enrollment not reported)	Sarepta Therapeutics, Inc.	No published scientific reports identified.
Pharmacokinetic Study in Cerebral Spinal Fluid After a Single Dose of AVI-4020	NCT00387283	Posted: 10/13/2006 Completed: 6/2009	1	Completed	AVI-4020 Injection	14 (target) 11 (actual)	Sarepta Therapeutics, Inc.	Press Release: https://investorrelations.sarepta.com/news-releases/news-release-details/avi-biopharma-announces-positive-clinical-trial-results
A Trial to Evaluate the Safety of a Single Intravenous Infusion of MGAWN1 in Healthy Adults	NCT00515385	Posted: 8/13/2007 Completed: 1/2009	1	Completed	MGAWN1 vs Placebo (normal saline)	40	MacroGenics, NIAID*	PMID: 20350945
Treatment of West Nile Virus With MGAWN1	NCT00927953	Posted: 6/25/2009 Completed: 5/2011	2	Terminated	MGAWN1 vs Placebo (normal saline)	13	MacroGenics, NIAID*	No published scientific reports identified.

*NIAID, National Institute of Allergy and Infectious Diseases.

WNV, West Nile virus.

One of the factors the authors of the Omr-IgG-am study cite as a barrier to enrollment was the prolonged period necessary to obtain Institutional Review Board (IRB) approval for each clinical site. Currently, each clinical site participating in a study must have independent IRB approval, a process that can take upwards of 6 months. This precludes the enrollment of any acute cases that present at a hospital or clinic which is not already approved. These authors argue that having one centralized IRB would have facilitated the timely enrollment of participants from a wide variety of clinical sites [78]. Additionally, the sporadic occurrence of many WNND cases can make it difficult to reach necessary target enrollment.

### Diagnostics

Several studies have documented the underdiagnosis of WNV [27,50,82], with barriers to diagnosis prevalent and varied. The majority of those infected experience a mild course of illness with few transient signs and symptoms or none at all (subclinical illness) [83]. There are few opportunities to diagnose these cases as their symptoms are not severe enough for them to seek medical care. These missed cases not only complicate the calculation of incidence and prevalence estimates, but also raised the concern for transmission via blood products provided by asymptomatic, viremic donors. In response to this concern, requirements for screening the US blood supply for viral RNA were initiated in in 2003 [26,27]. Public health surveillance of presumptive viremic blood donors has since become an important resource in determining the incidence of asymptomatic and subclinical infection [46,84].

Even clinically evident infections face diagnostic challenges. WNF is a loosely defined syndrome that resembles influenza and other viral syndromes [83,85]. A large study of blood donors in the US found that only 38% of subjects who were WNV positive and had symptoms sought medical care, and only 5% of those who did were formally diagnosed with WNV [27]. Patients with neuroinvasive manifestations (WNND) are more likely to receive a diagnosis than those with WNF, largely because the severity of signs and symptoms necessitates medical care and a definitive diagnosis is actively pursued. Clinical testing for WNV is estimated to occur in approximately 40% of WNV-compatible meningitis and encephalitis adult cases [50,51] and in approximately 25% of compatible pediatric cases [50]. Patients with an encephalitic presentation are more likely to be tested for WNV than those with meningitis, although meningitis is estimated to account for 30% to 50% of WNND cases [50]. The underdiagnosis of WNV is further illustrated in a multisite analysis of hospital-based WNV testing among meningitis and encephalitis patients. In this study, 84% of patients found by the study team to be WNV IgM positive in CSF received some WNV testing as part of their diagnostic workup, but 25% of cases who were tested clinically were missed due to inadequate testing [82].

Currently, the most widely used method for diagnostic WNV testing is detection of anti-WNV IgM antibodies in serum or CSF. This method has several important drawbacks. First, flaviviruses are known to produce antibodies which are cross-reactive with other flaviviruses, and it is therefore recommended that all diagnoses made in this manner are confirmed by sending acute and convalescent serum samples to a reference laboratory for plaque-reduction neutralization tests (PRNTs). This extra step must be performed in a Biosafety Level 3 (BSL-3) laboratory [86] which can be inconvenient and time-consuming. Alternatively, pseudotype viruses are available to use for PRNTs which would allow for an option to bypass the need for BSL-3 containment [87]. Second, IgM antibodies appear in serum between 3 and 8 days after symptom onset, so it is possible that early testing may provide a false negative result. Anti-WNV IgM antibodies can persist in serum for years after acute infection [88,89], with 1 study reporting detectable levels of IgM up to 8 years after infection in approximately 20% of participants [90]. Additionally, the currently available tests for detecting WNV IgM lack a high level of sensitivity, only 54% for ELISA and 45% for immunofluorescence assay (IFA), and measures of sensitivity and specificity may vary between viral lineages [91]. This greatly reduces the diagnostic value of this test in the acute setting, as it cannot be certain that the absence of IgM excludes a diagnosis of WNV or that its presence indicates acute infection.

Testing limitations have been an important consideration in ensuring the adequate screening of blood donations. Nucleic acid testing (NAT) of donor plasma has been used to screen blood donations in the US since 2003. Minipools consisting of multiple donor samples are typically tested first (MP-NAT), with reactive pools further investigated by individual donation testing (ID-NAT) of each sample included in the pool. As ID-NAT is more sensitive than MP-NAT, protocols are in place to trigger laboratories to switch to ID-NAT during high-WNV activity conditions. In the years following the implementation of these screening practices, the identification of a number of transfusion-related WNV transmissions sparked an interest in the distribution of virions within the different blood components. A 2007 study by researchers at the Food and Drug Administration (FDA) used reverse transcription polymerase chain reaction (RT-PCR) to assess viral load in the red blood cell (RBC) component versus the plasma component and found that viral load was one order of magnitude higher in the RBC component [92]. Others have continued this investigation and confirmed that WNV RNA is present in the RBC component for up to 3 months [93]. Interesting theories that have arisen from this research include that viral adherence to the RBCs occurs around the time of seroconversion [86] and that blood type may influence the ability of the virions to attach to proteins on the cell membrane [93]. Incorporating whole blood PCR with IgM testing may be critical to properly identify cases in a timely and cost-efficient manner in the future.

RT-PCR has been used to identify WNV RNA in other sample types, including CSF, urine, and saliva [82,94–97]. Testing for viral RNA in CSF seems to be of limited value, as some reports claim it is often undetectable around the time of symptom onset [82]. This may help explain why only 16.6% of samples from patients diagnosed with WNV through other methods were positive in CSF upon RT-PCR [97]. To date, only 1 study has examined the presence of WNV RNA in saliva. It was found in only 1 of 10 participants and persisted until approximately 9 days post-onset [95]. Several studies have examined the utility of testing for WNV RNA in urine [94,95,97], and although it appears to perform better than saliva and CSF, the best reported sensitivity estimate was 58.3% [97]. Regarding the testing of whole blood, the findings of these studies are consistent with the previously cited work. Whole blood was found to have a sensitivity of 86.8% [97], and a Weibull distribution model based on banked samples from the Houston West Nile Cohort showed that the majority of serial whole blood samples were positive for WNV RNA until approximately 3 months after symptom onset (50% negative at day 79 and 95% negative at day 119) [95].

### Becoming a neglected disease: Grant funding and publications over time

**Fig 3:**
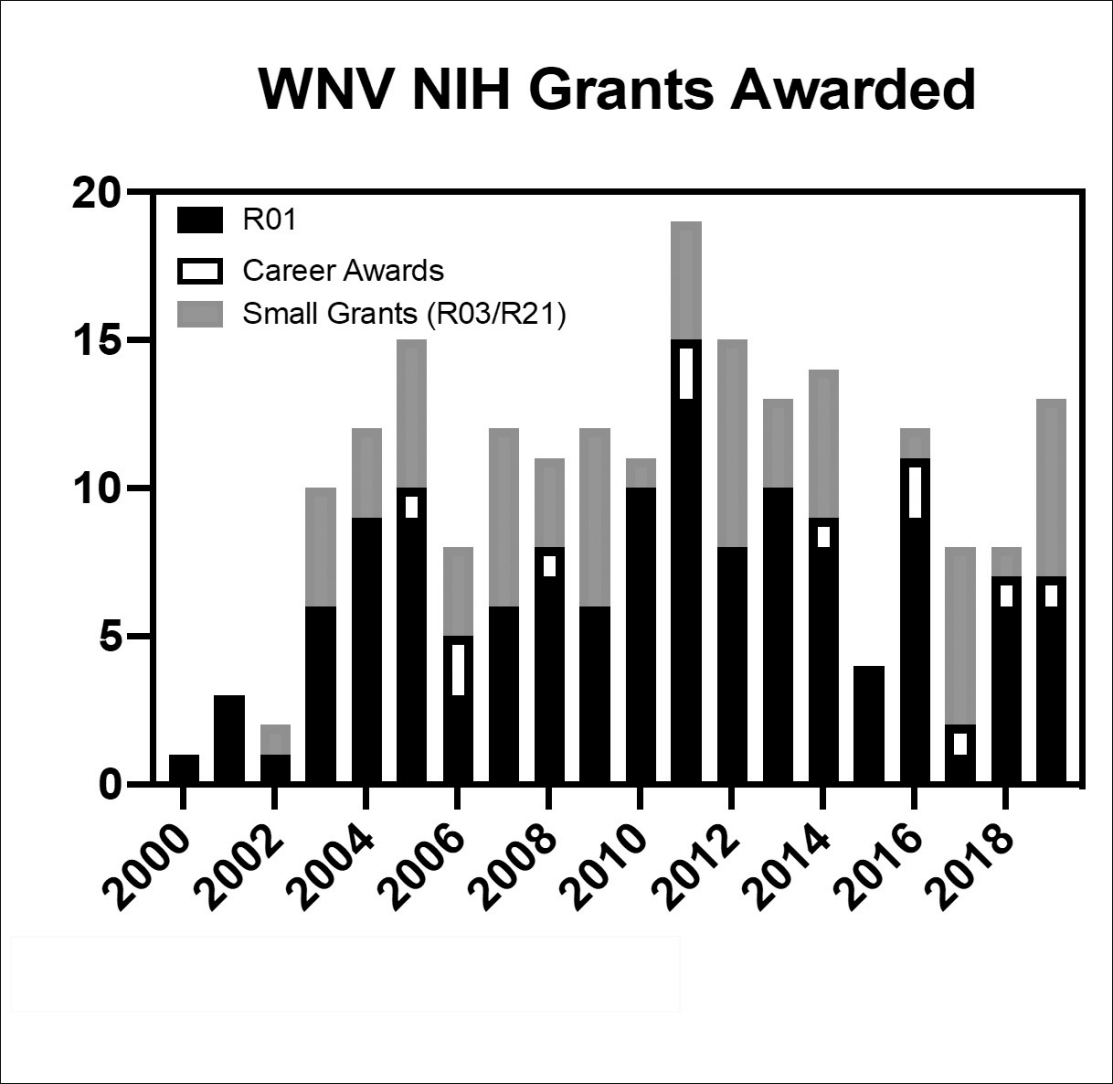
Number of NIH awards granted since 2000. NIH, National Institutes of Health.

For the last 2 decades, WNV has been discussed as an emerging infectious disease, but we and others find evidence to support its designation as a neglected disease. Although research has progressed, standards of care are still limited to supportive measures alone with no proven therapeutic targets or preventive vaccines. When grant funding directed toward these efforts from the National Institutes of Health (NIH) was evaluated using the RePORTER tool, approximately US$67 million dollars was directed to WNV-related research from 2000 to 2019 (approximately US$3.4 million per year), although some of this was dedicated to general research of the flaviviruses. The number of grant proposals funded by NIH per year since that time can also be recorded (Fig 3), with the majority of those grants being R01s, followed by the small grants such as R03s and R21s, with the least funding found in career development awards. Comparatively, over the same time period, NIH has awarded more than US$922 million for Zika virus research, with more than 99% of awards granted between 2016 and 2019 (approximately US$230 million per year).

**Fig 4:**
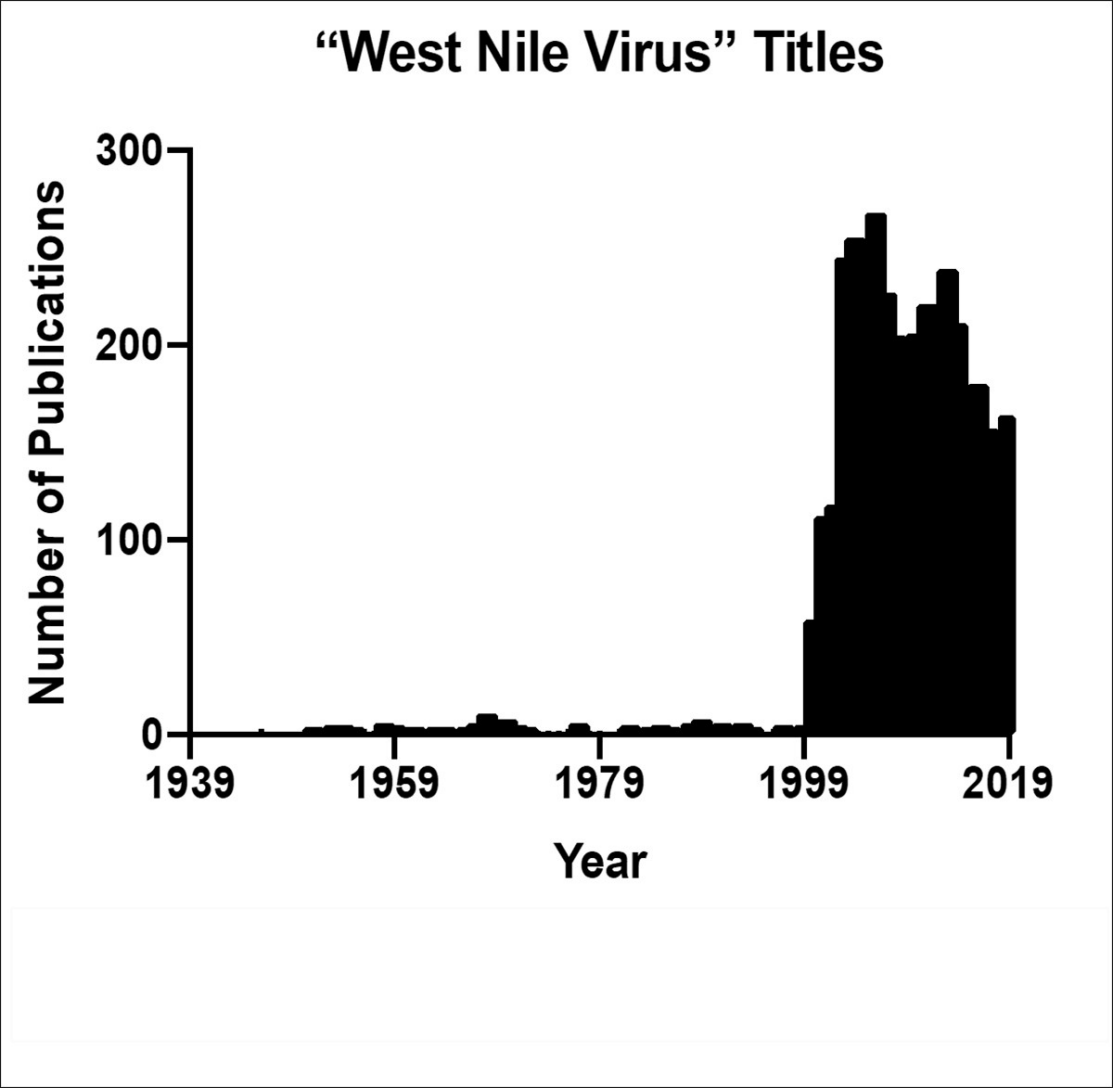
Trend of WNV publications over time. These reflect only those archived in PubMed. WNV, West Nile virus.

A PubMed search of “West Nile virus” research in the titles of indexed articles returns 3,978 articles (Fig 4). These articles span from 1946 through early November 2019 and highlight articles explicitly researching aspects of WNV, whether it be ecology, epidemiology, genetics, sequelae, therapeutic development, or other critical aspects of the disease process.

WNV has not only been widely researched in its own right, but also used as a tool or a guide to delineate mechanisms and outcomes of related flavivirus infections. In fact, if this PubMed search is expanded to include “West Nile virus” in both titles and abstract fields, one will find 6,229 results as of early November 2019. Many of these additional approximately 2,000 articles reference pathways or outcomes of WNV as a guiding principle for the research their teams sought to complete. Interestingly, when the publications of WNV are compared to another flavivirus—Zika virus—it is observed that approximately 3,600 articles mention Zika virus in the title from 2016 to 2019. The response effort to WNV, a disease that annually directly affects the US, has been less than that to other, more recently emerged and related infections.

## Conclusions

The arrival of WNV in the US was unprecedented and resulted in a reactive flurry of research, surveillance, and control measures. Despite these efforts, WNV is now endemic in North America, with nearly 7 million cases estimated to have occurred since 1999. True incidence and prevalence are difficult to determine, in part, because of inconsistent testing practices and inadequate testing methods. PCR testing of whole blood has demonstrated a higher sensitivity than IgM ELISA and should be further investigated as an alternative diagnostic tool. Infection causes significant morbidity and mortality each year, a costly medical and economic burden. Yet vaccine and therapeutic developments have progressed slowly in the 20 years since WNV first arrived in the US. Although an equine vaccine was approved shortly thereafter and has shown great success in almost eliminating equine cases and reducing risk of death by more than 40% [98], no human vaccine trials have proceeded past Phase II. Similarly, few clinical trials have tested therapeutic agents for WNV, and treatment is limited to supportive care. With sporadic epidemic activity, enrollment for these trials is challenging, and few options exist for increasing the number of participants across hospital systems throughout the US while maintaining human subject compliance. WNV may no longer be considered a novel or emerging pathogen in the US, but its clinical and public health significance has not diminished. Recent grant funding and publication volume have not reflected the continued high burden of WNV infection, while newer emerging pathogens with less burden have attracted greater attention. WNV has arguably become a neglected tropical disease based on the World Health Organization’s criteria for inclusion in category A [99] in that (1) it affects vulnerable populations, including those living in poor socioeconomic conditions with high morbidity and financial costs; (2) it affects those living in climates that are conducive to mosquito-borne diseases; (3) is amenable to control through comprehensive vector surveillance, source reduction, and adulticides to eliminate infectious female mosquitoes; and (4) the disease is inadequately addressed by clinicians, researchers, and policy makers. Future priorities should include research and product development for vaccines and antiviral and immunomodulating therapeutics.

**Key learning points**

- In the 20 years since West Nile virus (WNV) first emerged in the United States, more than 51,000 clinical cases have been reported, including more than 2,300 deaths, while an estimated 7 million people have been infected.
- Vaccine and therapeutic developments have progressed slowly over the past 20 years, with no licensed vaccine or therapeutic option approved for human use at this time.
- Recent grant funding and publication volume have not reflected the continued high burden of WNV infection, while newer emerging pathogens with less burden have attracted greater attention.
- WNV should be officially recognized as a neglected tropical disease since it meets the World Health Organization’s criteria for inclusion in category A.

**Top Five Papers**

1. Nash D, Mostashari F, Fine A, Miller J, O’Leary D, Murray K, et al. The outbreak of West Nile virus infection in the New York City area in 1999. N Engl J Med. 2001;344(24):1807–14. Epub 2001 Jun 16. doi: 10.1056/NEJM200106143442401. PubMed PMID: 11407341.
2. May FJ, Davis CT, Tesh RB, Barrett AD. Phylogeography of West Nile virus: from the cradle of evolution in Africa to Eurasia, Australia, and the Americas. J Virol. 2011;85(6):2964–74. doi: 10.1128/JVI.01963-10. PubMed PMID: 21159871; PubMed Central PMCID: PMC3067944.
3. Murray KO, Mertens E, Despres P. West Nile virus and its emergence in the United States of America. Vet Res. 2010;41(6):67. Epub 2010 Dec 29. doi: 10.1051/vetres/2010039. PubMed PMID: 21188801; PubMed Central PMCID: PMC2913730.
4. Staples JE, Shankar MB, Sejvar JJ, Meltzer MI, Fischer M. Initial and long-term costs of patients hospitalized with West Nile virus disease. Am J Trop Med Hyg. 2014;90(3):402–9. Epub 2014 Feb 12. doi: 10.4269/ajtmh.13-0206. PubMed PMID: 24515937; PubMed Central PMCID: PMC3945683.
5. Barber LM, Schleier JJ III, Peterson RK. Economic cost analysis of West Nile virus outbreak, Sacramento County, California, USA, 2005. Emerg Infect Dis. 2010;16(3):480–6. Epub 2010 Mar 6. doi: 10.3201/eid1603.090667. PubMed PMID: 20202424; PubMed Central PMCID: PMC3322011.
